# Chronic inflammation markers are associated with risk of pancreatic cancer in the Swedish AMORIS cohort study

**DOI:** 10.1186/s12885-019-6082-6

**Published:** 2019-08-29

**Authors:** Sam Sollie, Dominique S. Michaud, Debashis Sarker, Sophia N. Karagiannis, Debra H. Josephs, Niklas Hammar, Aida Santaolalla, Goran Walldius, Hans Garmo, Lars Holmberg, Ingmar Jungner, Mieke Van Hemelrijck

**Affiliations:** 1 King’s College London, School of Cancer and Pharmaceutical Sciences, Translational Oncology & Urology Research (TOUR), 3rd Floor, Bermondsey Wing, Guy’s Hospital London, London, SE1 9RT UK; 2 Department of Public Health and Community Medicine, Tufts University School of Medicine, Boston, MA USA; 3 Department of Epidemiology, Brown University School of Public Health, Providence, RI USA; 4 Department of Medical Oncology, Guy’s and St Thomas’ NHS Trust, London, UK; 5 St John’s Institute of Dermatology, School of Basics and Medical Biosciences, King’s College London, Guy’s Hospital, London, UK; 6 Unit of Epidemiology, Institute of Environmental Medicine, Karolinska Institutet, Stockholm, Sweden; 7 Unit of Cardiovascular Epidemiology, Institute of Environmental Medicine, Karolinska Institutet, Stockholm, Sweden; 8 Department of Medicine, Clinical Epidemiological Unit, Karolinska Institutet and CALAB Research, Stockholm, Sweden

**Keywords:** Chronic inflammation, Pancreatic cancer, CRP, Albumin, Haptoglobin, Leukocytes, AMORIS

## Abstract

**Background:**

Nested case-control studies examining the association between serum markers of chronic inflammation, focused on three specific biomarkers (CRP, IL-8 and TNF-α), and risk of pancreatic cancer have reported no associations. In this study, we evaluated associations between standard pre-diagnostic serum markers of chronic inflammation (CRP, albumin, haptoglobin and leukocytes) and pancreatic cancer risk in the Swedish Apolipoprotein-related MORtality RISk (AMORIS) prospective cohort study.

**Methods:**

We selected all participants (≥20 years old) with baseline measurements of CRP, albumin, haptoglobin and leukocytes between 1985 and 1996 (*n* = 61,597). Participants were excluded if they had a history of chronic pancreatitis and all individuals were free from pancreatic cancer at baseline. Cox proportional multivariable hazards regression analysis was carried out for medical cut-offs of CRP, albumin, haptoglobin and leukocytes.

**Results:**

We observed an increased risk of pancreatic cancer for those individuals with higher levels of serum haptoglobin (≥1.4 g/L), CRP (≥10 mg/L) and leukocytes (≥10 × 10^9^ cells/L) compared to those with haptoglobin levels < 1.4 g/L, CRP levels < 10 mg/L and Leukocyte levels < 10 × 10^9^ cells/L [haptoglobin HR: 2.23 (95% CI 1.72–2.88), CRP HR: 1.32 (95% CI 1.00–1.74), leukocytes HR: 2.20 (95% CI 1.52–3.18)]. No associations were noted for serum albumin.

**Conclusions:**

We found an increased risk of pancreatic cancer associated with pre-diagnostic serum levels of haptoglobin, CRP and leukocytes. Our finding suggests a possible role of chronic inflammation in the aetiology of pancreatic cancer and highlight the need to further investigate this association.

## Background

Apart from tobacco smoking, long-standing diabetes, obesity and chronic pancreatitis, more direct evidence for risk factors of pancreatic cancer remains to be established [1]. For many cancers, inflammation is a critical component of tumour progression [2]. Recently, mounting evidence points to chronic inflammation as a key mediator of pancreatic cancer development [3]. Two nested case-control studies in the Alpha-Tocopherol, Beta-Carotene Cancer Prevention (ATBC) Study and Prostate, Lung, Colorectal and Ovarian (PLCO) Cancer Screening trial found no association between pre-diagnostic circulating C-reactive protein concentrations and the risk of pancreatic cancer [4]. Another case-control study nested in the European Prospective Investigation into Cancer and Nutrition (EPIC) cohort did also not find an association between pre-diagnostic circulating CRP, interleukin-6 (IL-6), tumour necrosis factors (TNF-α) and pancreatic cancer risk [5]. In the nested case-control study from Health Professionals Follow-up study, Nurses’ Health Study, Physicians’ Health Study, Women’s Health initiative, an Women’s Health Study, no association was observed between pre-diagnostic circulating C-reactive protein (CRP), IL-6, TNF-α and pancreatic cancer risk [6]. Nevertheless, in several small hospital-based case-control studies, CRP concentrations were significantly higher in pancreatic cancer cases compared to chronic pancreatitis patients or controls [7–9]. Other common serum markers of inflammation such as haptoglobin, leukocytes and albumin, are less well studied in relation to the risk of pancreatic cancer even though they have been found to be associated with other malignancies [10–12]. A small study at the Royal Infirmary of Edinburgh, including 42 pancreatic cancer patients and 12 controls, observed a statistically significant lower serum albumin levels in pancreatic cancer patients compared to controls [8]. In addition to these biomarker studies, it is worth noting that chronic pancreatitis (CP), a progressive inflammatory process that results in the permanent damage of the organ structure, is associated with a 13.3-fold risk of pancreatic cancer and up to 33-fold risk in patients who suffer from both diabetes and CP [13, 14]. However, few serum markers of chronic inflammation have been investigated in relation to CP and pancreatic cancer diagnosis (mainly CRP and cytokines such as Interleukin-6 (IL-6) and Tumour Necrosis Factors (TNF-α)), partially because CP may elevate pancreatic enzymes instead [5, 15–17].

Better understanding causes and underlying biological mechanisms for pancreatic cancer may improve our ability to identify high risk individuals and improve early detection. The current study aimed to evaluate associations between standard pre-diagnostic serum markers of chronic inflammation (CRP, albumin, haptoglobin and leukocytes) and pancreatic cancer risk in the prospective Swedish Apolipoprotein-related MORtality RISk (AMORIS) cohort study. This is the first prospective cohort study to examine common serum markers of chronic inflammation in relation to pancreatic cancer.

## Methods

### Study population and data collection

The Swedish Apolipoprotein-related MORtality RISk (AMORIS) cohort includes information from blood and urine samples for 812,073 subjects obtained between 1985 and 1996 on a number of biomarkers. All laboratory analyses were done at the Central Automation Laboratory (CALAB), Stockholm. The subjects were residents of Sweden and were predominantly living in Stockholm county, ranging in age from less than 20 to over 80 years old. All participants were either healthy individuals referred for clinical laboratory testing as part of health check-ups or outpatients referred for laboratory testing. A more detailed description of the AMORIS cohort is given elsewhere [18–22].

The AMORIS cohort has been followed via record linkage using the Swedish 10-digit personal identity number in Swedish national health registers, registers of quality of care, and surveys including socio-economic data as well as questionnaire and biomedical data from number of research cohorts [15]. For the purpose of the current study, we used information from the National Cancer Register, the Patient Register, the Cause of death Register and the consecutive Swedish Censuses during 1970–1990. This study complied with the Declaration of Helsinki and was approved by the Ethics Review Board of the Karolinska Institute.

We included all individuals aged 20 years or older who were free from pancreatic cancer at baseline, as registered in the National Cancer Register going back to 1958. Furthermore, individuals were excluded if they had a history of chronic pancreatitis, as defined in the National Patient Register going back nationally to 1987 and regionally to 1964. All subjects were required to have baseline measurements of CRP, albumin, leukocytes and haptoglobin available from the same health examination between 1985 and 1996. If a participant had multiple measurements of a serum marker of chronic inflammation, the first measurement was included in the study (*n* = 61,597).

Follow-up time was defined as time from baseline measurement until the date of cancer diagnosis, death, emigration, or end of the study (31st of December 2011), whichever occurred first.

The outcome investigated in this study was a diagnosis of pancreatic cancer (International Classification of Diseases (ICD), Revision 7 (1955) code 157). We also included the following information from the AMORIS study: serum CRP (mg/L), albumin (g/L), leukocytes (10^9^ cells/L), haptoglobin (g/L), age at baseline measurement and gender. From the other registries, we collected information regarding education, comorbidities coded following the Charlson Comorbidity Index (CCI) [23] and cancer diagnosis. Serum glucose (mmol/L) levels were also obtained given that diabetes is a risk factor for pancreatic cancer and is also associated with inflammation [14, 24, 25].

The sensitive quantitative method used for the determination of serum CRP and haptoglobin was an immunoturbidimetric assay (reagents from Orion Diagnostics, Espoo, Finland) using fully automated multichannel analyses (for CRP an AutoChemist – PRISMA, New Clinicon, Stockholm, Sweden 1985–1992 and a DAX 96, Technicon Instruments, Corporation, Tarrytown, NY, USA, 1993–1996; for the measurement of haptoglobin Hitachi-analysers, Boehringer Mannheim, Baden-Wurttemberg, Germany) were performed. The measurement of high sensitivity CRP was not available during the period of blood sample collection (1985–1996). Therefore, CRP levels < 10 mg/L could not be measured precisely and the 10 mg/L cutoff has been used in the study. However, that cutoff is broadly accepted as the upper limit of the clinical reference range. The sensitive quantitative method used for the determination of serum albumin was the bromocresol green method. Leukocytes measurements were performed using hematology analyzers (STKS Haematology System from Coulter Corporation, Hialeah, FL). Total imprecision calculated by the coefficient of variation was 12% at CRP level 40 mg/L, 5.6% at haptoglobin level 1.1 g/L, < 1.8% for albumin and < 2.7% at leukocytes 10 X 10^9^ cells/L [26].

### Data analyses

We estimated the risk of pancreatic cancer with multivariate Cox proportional hazards regression analysis for medical cut-offs used in the CALAB laboratory for CRP: < 10 mg/L and ≥ 10 mg/L; haptoglobin: < 1.4 g/L and ≥ 1.4 g/L; leukocytes: < 10 10^9^ cells/L and ≥ 10 10^9^ cells/L [27]. Albumin was dichotomised as < 40 g/L and ≥ 40 g/L instead of the medical cut-off of 35 g/L due to the small number of participants with low albumin levels [28]. Cox proportional hazards regression models were adjusted for age, gender, education, CCI and serum glucose levels. We conducted a sensitivity analysis in which those who had a follow-up time < 1 year and < 3 years respectively were removed, to assess potential role of reverse causation.

With regards to haptoglobin, CRP and leukocytes, we additionally performed stratified analyses for age (< 55 & ≥55), gender (male & female) and serum glucose levels (< 7.00 mmol/L & ≥7.00 mmol/L). A *P*-value for interaction was also calculated.

All statistical analyses were conducted with Statistical Analysis Systems (SAS) release 9.4 (SAS Institute, Cary, NC).

## Results

Characteristics of study participants are shown in Table 1. During a mean follow-up of 18.3 years, 286 participants developed pancreatic cancer. The mean age in participants who later developed pancreatic cancer was higher (59.8) than in participants without pancreatic cancer (50.0). In subjects with a diagnosis of pancreatic cancer during follow-up, there were more women than men (54.5% vs. 45.5%).

**Table 1:** Descriptive statistics of study population

	Pancreatic cancer *N* = 286 n (%)	No pancreatic cancer *N* = 61,311 n (%)
Mean Age (years) (SD)	59.8 (12.01)	50.0 (15.94)
< 55	101 (35.3)	38,417 (62.7)
≥ 55	185 (64.7)	22,894 (37.3)
Gender		
Men	130 (45.5)	26,717 (43.6)
Women	156 (54.5)	34,594 (56.4)
SES		
Unclassified/Missing	53 (18.5)	9701 (15.8)
Low	99 (34.6)	24,282 (39.6)
High	134 (46.9)	27,328 (44.6)
Education		
Missing	39 (13.6)	4213 (6.9)
Low	68 (23.8)	14,428 (23.5)
Middle	104 (36.4)	25,240 (41.2)
High	75 (26.2)	17,430 (28.4)
Comorbidities		
0	253 (88.5)	56,346 (91.9)
1	27 (9.4)	3476 (5.7)
2	3 (1.0)	858 (1.4)
3+	3 (1.0)	631 (1.0)
Mean follow-up time (years) (SD)	10.2 (6.49)	18.3 (5.53)
Serum glucose (mmol/L)		
Mean (SD)	5.55 (2.02)	5.06 (1.45)
< 5.6 mmol/L	197 (68.9)	50,219 (81.9)
5.6–6.9 mmol/L	58 (20.3)	7281 (11.9)
≥ 7 mmol/L	24 (8.4)	2511 (4.1)
Missing	7 (2.4)	1300 (2.1)
Albumin (g/L)		
Mean (SD)	41.73 (2.83)	42.51 (2.90)
< 40 g/L	55 (19.2)	8678 (14.2)
≥ 40 g/L	231 (80.8)	52,633 (85.8)
Haptoglobin (g/L)		
Mean (SD)	1.20 (0.35)	1.08 (0.32)
< 1.4 g/L	199 (69.6)	51,885 (84.6)
≥ 1.4 g/L	87 (30.4)	9426 (15.4)
C-reactive protein (mg/L)		
Mean (SD)	6.78 (10.74)	5.56 (14.51)
< 10 mg/L	217 (75.9)	50,551 (82.5)
≥ 10 mg/L	69 (24.1)	10,760 (17.5)
Leukocytes (10^9^ cells/L)		
Mean (SD)	7.42 (2.94)	6.67 (2.29)
< 10 10^9^ cells/L	253 (88.5)	57,330 (93.5)
≥ 10 10^9^ cells/L	33 (11.6)	3981 (6.5)

Multivariate Cox regression analysis (adjusted for age, gender, education, CCI and serum glucose level) for the association between markers of chronic inflammation and risk of pancreatic cancer showed a positive association with risk of pancreatic cancer for those with higher levels of serum haptoglobin (≥1.4 g/L) compared to those with haptoglobin levels < 1.4 g/L [HR: 2.23 (95% CI 1.72–2.88)]. We also observed a borderline significant positive association with risk of pancreatic cancer for those with higher levels of CRP (≥10 mg/L) compared to those with CRP levels < 10 mg/L [HR: 1.32 (95% CI 1.00–1.74)]. Furthermore, we observed a positive association with risk of pancreatic cancer for those with higher levels of leukocytes (≥10 × 10^9^ cells/L) compared to those with leukocyte levels < 10 × 10^9^ cells/L [HR: 2.20 (95% CI 1.52–3.18)] (Table 2). No association was observed for albumin. A sensitivity analysis to assess reverse causation by excluding those with follow-up time < 1 year and < 3 year did not affect the above findings substantially (results not shown).

**Table 2:** Hazard ratio (HR) for risk of pancreatic cancer with 95% confidence intervals (CI) using Cox proportional hazards models

	Pancreatic cancer/Total N	Hazard Ratio^a^ (95% CI)
Albumin (g/L)		
< 40 g/L	55/8733	1.11 (0.82–1.50)
≥ 40 g/L	231/52,864	1.00 (Ref)
Haptoglobin (g/L)		
< 1.4 g/L	199/52,084	1.00 (Ref)
≥ 1.4 g/L	87/9513	2.23 (1.72–2.88)
C-reactive protein (mg/L)		
< 10 mg/L	217/50,768	1.00 (Ref)
≥ 10 mg/L	69/10,829	1.32 (1.00–1.74)
Leukocytes (10^9^ cells/L)		
< 10 10^9^ cells/L	253/57,583	1.00 (Ref)
≥ 10 10^9^ cells/L	33/4014	2.20 (1.52–3.18)

^a^ Adjusted for age, gender, education, CCI and serum glucose (continuous variable)

We performed a stratified analysis for age, gender and glucose levels, but no effect modification was observed (results not shown).

## Discussion

In this study, by interrogating serum data from 61,597 healthy subjects in the AMORIS cohort with follow-up of 18 years, we found evidence for a positive association between serum haptoglobin, CRP and leukocytes, and the risk of developing pancreatic cancer. No association was found between serum albumin and the risk of pancreatic cancer.

Inflammation is a critical component of tumour development and progression [2, 29, 30]. There is increasing evidence for the role that local immune response and systemic inflammation may play in tumour progression [31]. Known cancer types related to chronic inflammation are: *Helicobacter Pylori*-associated gastric cancer, hepatocellular carcinoma and inflammatory bowel disease-associated colorectal cancer [30]. Pancreatic cancer has only in the past two decades been recognised as an inflammation-driven cancer [32]. Smoking, obesity, and diabetes, all established risk factors of pancreatic cancer, may increase risk by causing systemic inflammation. On the other hand, chronic pancreatitis, another well-known risk factor for pancreatic cancer presents with slow subclinical chronic inflammation of the pancreas [13, 14]. Epidemiological data suggest that *Helicobacter pylori and Porphyromonas gingivalis* play a role in pancreatic carcinogenesis. Infection due to these bacteria may also lead to elevated markers of systemic inflammation [33].

However, despite the evidence for a link between inflammation and pancreatic cancer, the inflammatory mediators that may promote pancreatic cancer development remain poorly defined and studies to date are limited to three acute-phase inflammatory factors: CRP, IL-6 and TNF-α, which show no clear associations [4–6].

To our knowledge, this is the first prospective cohort study assessing associations between standard pre-diagnostic serum markers of chronic inflammation and the risk of developing pancreatic cancer. We found an increased risk of developing pancreatic cancer when participants have increased levels of haptoglobin, CRP and leukocytes, serum markers of inflammation. This indeed supports the notion that pancreatic cancer is an inflammation driven cancer [32]. We found a borderline significant positive association between CRP and risk of pancreatic cancer, this finding is different from previous case-control studies about this association [4–6].

Differences in study design may explain this different result. To our knowledge, the previous studies (EPIC, ATBC, PLCO & U.S. cohorts) did not exclude participants with chronic pancreatitis or other comorbidities, apart from diabetes [4–6]. Moreover, even though all the markers play a role in the inflammatory cascade, the roles and mechanisms of action are diverse between the different molecules which could explain why IL-6 and TNF-α (reported to play a role in the induction of the CRP cascade) show a null result in comparison with haptoglobin and leukocytes [8, 34–37].

The major strength of this study is the large number of prospective measurements of serum markers of chronic inflammation in the AMORIS cohort, all measured at the same clinical laboratory which have used internationally accredited and calibrated methods [26]. The database provided complete follow-up for each participant as well as linkage to other registers allowing for information about cancer status, death or emigration. All participants of the AMORIS cohort were selected by analysing blood and/or urine samples from health check-ups in non-hospitalized persons [38]. However, any healthy cohort effect would not affect the internal validity of our study. Our analyses contained more women than men, which is likely due to the higher likelihood of assessment of chronic inflammation markers in women as part of a pregnancy-related health check-up. Sex was treated as a confounder and an effect modifier in the analyses. It was also a limitation that high-sensitive CRP was not available at the time measurements were conducted in CALAB. CRP levels < 10 mg/L were unquantifiable, which may have resulted in an underestimation of the association with risk of pancreatic cancer. The biomarkers Interleukin-6 and tumour necrosis factors, other commonly used markers of inflammation, were not available in the AMORIS cohort. In addition, there were not enough repeated measurements to verify the timeline between changes in markers of chronic inflammation and risk of pancreatic cancer. We did not have information on other possible confounders such as BMI and smoking status, which may have impacted our findings. However, all models were adjusted for the Charlson Comorbidity Index.

## Conclusion

This is the first prospective cohort study evaluating the association between standard pre-diagnostic serum markers of chronic inflammation and the risk of pancreatic cancer. We observed a positive association between haptoglobin, CRP and leukocytes and the risk of pancreatic cancer. These findings suggest the importance of inflammation as one of the underlying mechanisms in carcinogenesis and suggests a role in the aetiology of pancreatic cancer. Future research should use other markers of chronic inflammation and repeated measurements to provide further insights into these associations.

## Data Availability

Access to data for collaboration is provided by the Steering group members of the AMORIS study by request in email under the heading AMORIS Cohort Collaboration. This can be found at the AMORIS homepage http://amoriscohort.imm.ki.se.
